# *Aphyllorchis yachangensis* (Orchidaceae), a new holomycotrophic orchid from China

**DOI:** 10.3897/phytokeys.179.63994

**Published:** 2021-07-05

**Authors:** Ying Qin, Hailing Chen, Zhenhai Deng, Yan Liu

**Affiliations:** 1 Guangxi Institute of Botany, Guangxi Zhuang Autonomous Region and Chinese Academy of Sciences, Guilin, Guangxi, 541006, China Guangxi Institute of Botany, Guangxi Zhuang Autonomous Region and Chinese Academy of Sciences Guilin China; 2 Management Center of Yachang Orchid National Nature Reserve, Baise, Guangxi, 533200, China Management Center of Yachang Orchid National Nature Reserve Baise China

**Keywords:** *
Aphyllorchis
*, Guangxi Province, new taxa, saprophytic Orchidaceae, taxonomy

## Abstract

*Aphyllorchis yachangensis*, a new holomycotrophic orchid from Guangxi, southern China is described and illustrated here. This new species is similar to *A. caudata* but differs from the latter mainly by the sepals acute at the apex, the hypochile with 2 smaller and semicircular wings, the epichile adaxially smooth, acute, the lateral lobes triangular-ovate and the column clavate.

## Introduction

*Aphyllorchis*Blume (1825) includes about 30 species, and is mainly distributed in tropical Asia and the Himalayas, extending to the north of Japan and to the south of Australia (Chen and Stephan 2009; Tian et al. 2013). The species of *Aphyllorchis* are holomycotrophic herbs, with short rhizomes; fleshy roots; erect, unbranched stems, with sheaths; racemose inflorescences; petals similar to sepals, free; lip 2-partite, hypochile smaller than epichile, sometimes without hypochile; staminodes 2; pollinia 2, granular-farinaceous, caudicles absent (Chen and Stephan 2009). In China, six species and one variety are reported from the South, Southwest and Taiwan Island, including *Aphyllorchis alpina* King & Pantling, *A. caudata* Rolfe ex Downie, *A. gollanii* Duthie, A. montana H. G. Reichenbach var. montana, A. montana H. G. Reichenbach var. membranacea T. C. Hsu, *A. pallida* Blume and *A. simplex* Tang & F. T. Wang (Chen and Stephan 2009; Fan et al. 2011; Hsieh et al. 2013; Huang et al. 2014; Hsu and Chung 2017; Lin 2017).

In late July 2018, during a botanic expedition in the Yachang Orchid National Nature Reserve, Guangxi Province in China, the first author collected a specimen (*Ying Qin* et al. *YC3639*) belonging to *Aphyllorchis*, with residual flowers. The narrow hypochile with 2 unobvious wings at base and purple epichile of the species drew the author’s attention. However, due to the incomplete structure of sepals, petals and column, it was not accurately identified at that point. Fortunately, after two further botanic expeditions, one in July 2019 and the other in July 2020, we successfully collected two specimens (*Ying Qin* et al. *QY20190719001* and *Ying Qin* et al. *QYYC20200703009*) belonging to the same taxon, but on these occasions with complete flowers. After dissecting and examining those flowers, and consulting the relevant literature (Reichenbach 1876; Downie 1925; Fukuyama 1934; Pearce and Cribb 1999; Roy et al. 2009; Aravindhan et al. 2013; Merckx et al. 2013; Tian et al. 2013; Campbell 2014; Sun et al. 2017; Suetsugu et al. 2018), we finally confirmed that it is a new species, which is described here.

## Material and methods

From July 2018 to July 2020, we examined four specimens of *Aphyllorchis* in IBK on the field, and also examined forty specimens of *Aphyllorchis* in PE, HITBC, KUN, AU, IMDY, SZG, etc. through CVH (https://www.cvh.ac.cn/index.php). Except for *Ying Qin* et al. *YC3639*, *Ying Qin* et al. *QY20190719001* and *Ying Qin* et al. *QYYC20200703009* collected by the first author and kept in IBK, none of the specimens belong to *Aphyllorchis yachangensis*. Photographs of plants and flowers were taken using a Canon PowerShot G16. Morphological characters of the new species were measured with a ruler on living plants in the wild. The terminologies used to describe parts of the new species, such as rhizome, ovary, sepals, petals, lip, hypochile, epichile, column, etc. come from Flora of China (Chen and Stephan 2009).

## Taxonomic Treatment

### Aphyllorchis yachangensis

Taxon classificationPlantaeAsparagalesOrchidaceae

Ying Qin & Yan Liu sp. nov.

AF8E39B6-8556-55B6-8975-809069C885A8

urn:lsid:ipni.org:names:77218063-1

Figures 1 , 2

#### Diagnosis.

*Aphyllorchis yachangensis* is similar to *A. caudata* but differs from the latter mainly by its hypochile with two wings of 1–1.4 × 0.4–0.6 mm (vs. 2–3 × ca. 4 mm), epichile adaxially smooth (vs. densely papillose), lateral lobes triangular-ovate (vs. semicircular), sepals acute at apex (vs. long cuspidate) and column clavate (vs. approximately cylindrical). Detailed morphological comparisons between *A. yachangensis* and *A. caudata* are provided in Table 1.

**Table 1.** Morphological comparison of *Aphyllorchis yachangensis* and *A. caudata*.

Characters	*A. yachangensis*	*A. caudata*
Plant size	107–113 cm	100 cm
Inflorescence	42–47 cm	rachis to 50 cm
Bracts	lanceolate, **12–30** × 2.8–6.8 mm	narrowly lanceolate, **40–48** × 5–6 mm
Dorsal sepal	lanceolate, 25–40 × 7.9–10.7 mm, **apex acute**	linear-lanceolate or lanceolate, 30–35 × ca. 8.0 mm, **apex long cuspidate**
Lateral sepals	lanceolate, 6.1–8.2 mm wide, **apex acute**	linear-lanceolate or lanceolate, 6–7 mm wide, **apex long cuspidate**
Petals	lanceolate, **22–27** × 5.6–7.2 mm	lanceolate, **ca. 20** × 6–7 mm
Hypochile	**6.6–7.1** mm long, with 2 wings; **wings semicircular, 1–1.4 × 0.4–0.6 mm**	**2–3** mm long, with 2 wings; **wings ligulate, 2–3 × ca. 4 mm**
Epichile	**1.9–2.2** cm long, **adaxially smooth**; **lateral lobes triangular-ovate**	**ca. 1.2** cm long, **adaxially densely papillose; lateral lobes semicircular**
Column	1.4–1.6 cm long, **clavate (apex obviously inflated)**	1.1–1.4 cm long, **approximately cylindrical**
Flowering period	late June and July	July and August

**Figure 1.**
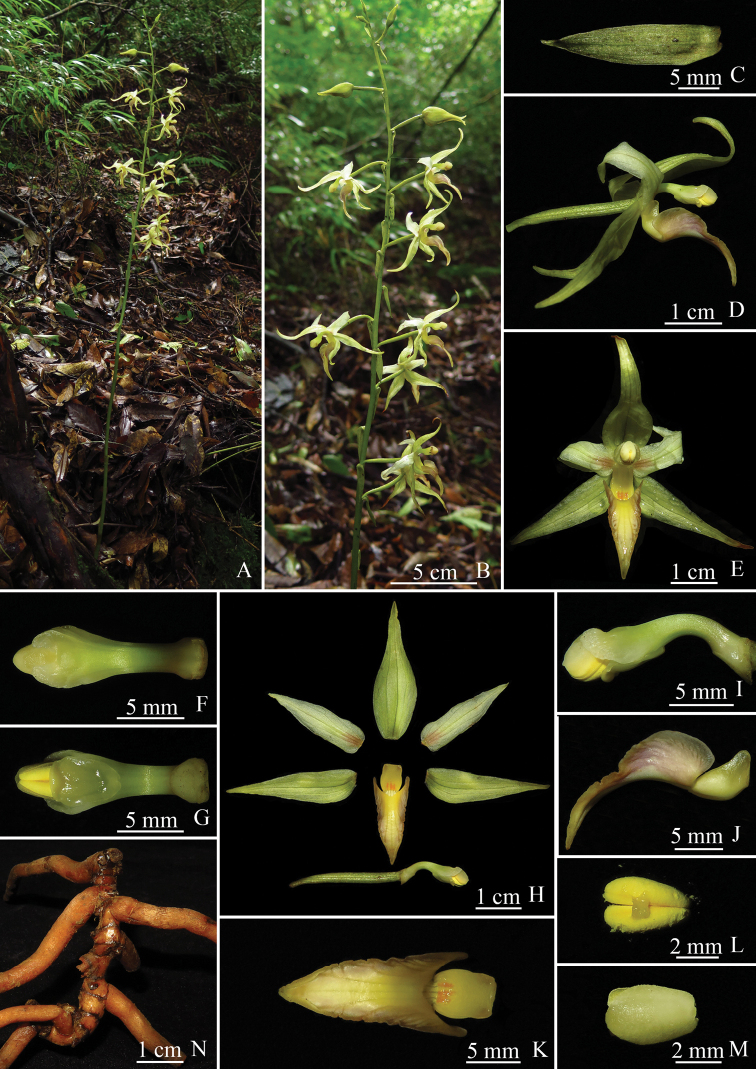
*Aphyllorchis yachangensis***A** flowering habit **B** inflorescence **C** bract **D** flower in side view **E** flower in front view **F** column in top view **G** column in ventral view **H** lip, dorsal sepal, lateral sepals, petals, column and ovary **I** column in side view **J** lip in side view **K** lip in top view **L** pollinaria **M** anther cap in top view **N** rhizome and roots. Photographed by Ying Qin from *Ying Qin* et al. *QY20190719001* (holotype: IBK!).

#### Type.

China. Guangxi, Baise city, Leye county, Yachang Orchid National Nature Reserve, 1862 m, 19 July 2019, *Ying Qin* et al. *QY20190719001* (***holotype***: IBK! isotype: IBK!).

#### Description.

Herb holomycotrophic, leafless, 107–113 cm tall. Rhizome ca. 10.8 cm long, 4.9–6.2 mm in diameter, densely noded; internodes 2.4–12 mm long. Roots spreading, stout, 3.4–6.4 mm in diameter. Stem 61–71 cm tall, 4.5–7.3 mm in diameter, yellow-green, usually with many dark purple stripes and spots, with 1.4–4 cm long sheaths; the uppermost sheath lanceolate; other sheaths tubular. Inflorescence terminal, racemose, 42–47 cm long, with 19–23 well-spaced flowers; rachis sparsely glandular puberulent, yellow-green, usually with many dark purple stripes and spots. Bracts lanceolate, shorter than pedicel and ovary, 1.2–3 cm long, 2.8–6.8 mm wide, yellow-green, usually with many dark purple stripes and spots on abaxial surface, sparsely glandular puberulent. Ovary cylindrical, yellow-green, usually with many dark purple stripes and spots, sparsely glandular puberulent, 2.2–2.8 mm in diameter, including pedicel to 2.3–3.5 cm long. Sepals yellow-green, usually with many dark purple stripes and spots on abaxial surface, abaxially sparsely glandular puberulent; dorsal sepal lanceolate, slightly concave, 2.5–4 cm long, 7.9–10.7 mm wide, apex acute, reflexed; lateral sepals lanceolate, slightly oblique at base, 2.5–4 cm long, 6.1–8.2 mm wide, apex acute, reflexed. Petals lanceolate, reflexed, 2.2–2.7 cm long, 5.6–7.2 mm wide, yellow-green, pale purple to dark purple at base, apex acute. Lip 2-partite; hypochile narrow, concave, 6.6–7.1 mm long, 3.6–4.5 mm wide, yellow or reddish brown, with 2 unobvious wings at base; wings semicircular, 1–1.4 mm wide at base, 0.4–0.6 mm tall; epichile ca. 1.9–2.2 cm long, 1.3–1.5 cm wide when flattened, 3-lobed, yellow with pale purple, or purple to dark purple, adaxially smooth, margin slightly erose; lateral lobes triangular-ovate, 8.2–8.9 mm long, 3.6–4.3 mm wide; mid-lobe narrowly triangular, 1.1–1.3 cm long, 5.6–5.9 mm wide, acute at apex, margin slightly involute. Column clavate, arcuate, 1.4–1.6 cm long, 1.8–3.3 mm in diameter in lower part, 4.3–6.1 mm in diameter in upper part, yellow-green, usually purple from middle to base; stigma ovate, slightly concave; staminodes 2, on both sides of apex, yellow-white to pale yellow-green; pollinia 2, ovate, granular-farinaceous, 4 mm long, yellow; anther cap ovate, slightly laterally compressed, 4 mm long, pale yellow.

**Figure 2.**
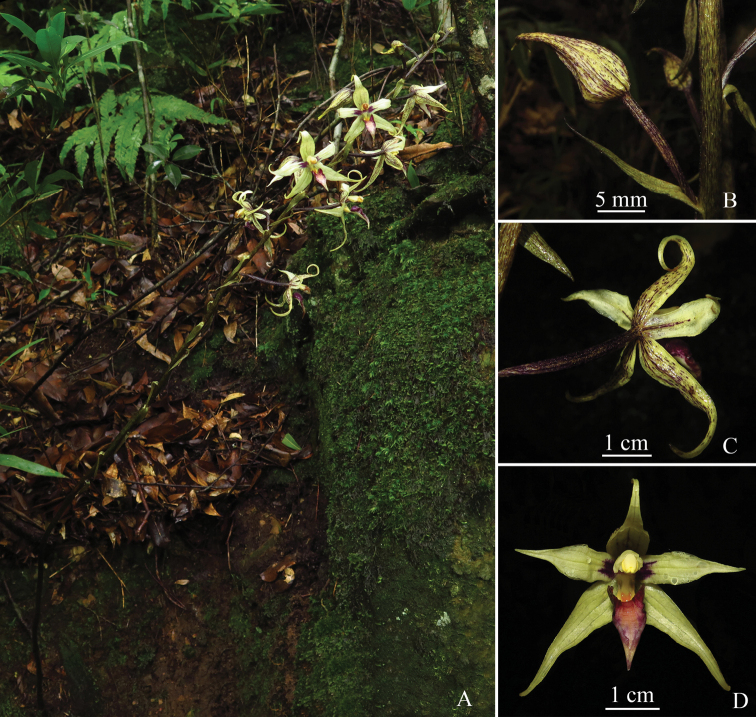
*Aphyllorchis yachangensis* (an individual with dark colors in the same environment with *Ying Qin* et al. *QY20190719001*) **A** flowering habit **B** bud **C** flower in back view **D** flower in front view. Photographed by Ying Qin from *Ying Qin* et al. *QYYC20200703009* (paratype: IBK!).

#### Distribution, habitat and ecology.

*Aphyllorchis yachangensis* was discovered in the Yachang Orchid National Nature Reserve, Leye county, Baise city, Guangxi Province, China. The holotype subpopulation is distributed in a subtropical evergreen and deciduous broad-leaved mixed forest, and is found growing with *Chimonobambusa quadrangularis* (Franceschi) Makino (Gramineae), *Lithocarpus glaber* (Thunberg) Nakai (Fagaceae), *Manglietia fordiana* Oliver (Magnoliaceae), *Juglans mandshurica* Maximowicz (Juglandaceae), *Dysosma majorensis* (Gagnep.) Ying (Berberidaceae), *Ophiopogon bockianus* Diels (Liliaceae), *Disporopsis fuscopicta* Hance (Liliaceae), *Calanthe brevicornu* Lindley (Orchidaceae), *Goodyera biflora* (Lindley) J. D. Hooker (Orchidaceae), *G. velutina* Maximowicz ex Regel (Orchidaceae), etc.

#### Phenology.

Flowering in late June and July, capsules not seen.

#### Etymology.

The specific epithet is derived from the type locality, Yachang Orchid National Nature Reserve.

#### Chinese name.

雅长无叶兰 (Ya Chang Wu Ye Lan)

#### Additional specimens examined

**(paratypes).** China: Guangxi: Baise city, Leye county, Yachang Orchid National Nature Reserve, 1862 m, 28 July 2018, *Ying Qin* et al. *YC3639* (IBK!); Guangxi: Baise city, Leye county, Yachang Orchid National Nature Reserve, 1859 m, 03 July 2020, *Ying Qin* et al. *QYYC20200703009* (IBK!).

### Key to *Aphyllorchis* taxa in China

**Table:** 

1	Lip similar to lateral petals	***A. simplex***
–	Lip distinctly different from petals	**2**
2	Bracts longer than pedicel and ovary	**3**
–	Bracts shorter than pedicel and ovary	**4**
3	Flowers yellowish green; bracts linear to linear-lanceolate, 3–4 mm wide; lip contracted at middle into hypochile and epichile	***A. alpina***
–	Flowers pale purplish brown; bracts ovate to elliptic-lanceolate, 6–8 mm wide; lip contracted near base into hypochile and epichile	***A. gollanii***
4	Sepals equal to or longer than 25 mm	**5**
–	Sepals equal to or shorter than 11 mm	**6**
5	Sepals apex long cuspidate; epichile adaxially densely papillose	***A. caudata***
–	Sepals apex acute; epichile adaxially smooth	***A. yachangensis***
6	Sepals 4–5 mm long	***A. pallida***
–	Sepals 9–11 mm long	**7**
7	Lip fleshy	**A. montana var. montana**
–	Lip membranous	**A. montana var. membranacea**

## Supplementary Material

XML Treatment for Aphyllorchis yachangensis
